# Preventing gambling‐related harm in adolescents (PRoGRAM‐A), a secondary school‐based social network intervention: Results from a pilot cluster randomised controlled trial

**DOI:** 10.1111/add.70267

**Published:** 2025-12-12

**Authors:** Fiona Dobbie, Martine Miller, Angela Niven, Heather Wardle, Christopher Weir, Hannah Ensor, Andrew Stoddart, Dave Griffiths, Leon Noble, Richard Purves, James White

**Affiliations:** ^1^ Usher Institute University of Edinburgh Edinburgh UK; ^2^ School of Social and Political Sciences University of Glasgow Glasgow UK; ^3^ Edinburgh Clinical Trials Unit University of Edinburgh Edinburgh UK; ^4^ Faculty of Social Sciences University of Stirling Stirling UK; ^5^ School of Earth and Environment University of Leeds Leeds UK; ^6^ Institute for Social Marketing University of Stirling Stirling UK; ^7^ Centre for Trials Research, School of Medicine Cardiff University Cardiff UK

**Keywords:** gambling, gambling related harm, peer education, public health, school‐based intervention, young people

## Abstract

**Aim:**

To conduct a pilot cluster randomised controlled trial (cRCT) of a gambling prevention intervention (PRoGRAM‐A) among young people aged 13–15 to determine the utility of conducting a Phase III RCT assessing effectiveness and cost‐effectiveness.

**Design:**

Two‐arm, pilot cluster randomised controlled trial with an embedded process evaluation, health economic scoping study and social network analysis. Six schools were identified based on Scottish Index of Multiple Deprivation and school roll size. Schools were randomised to either intervention (*n* = 4) or control (*n* = 2). The trial was delivered between October 2023 and November 2024.

**Setting:**

Six state funded secondary schools in Scotland (four intervention, two control).

**Participants:**

Students (intervention *n* = 762, and control *n* = 352) in secondary school year 3 (aged 13–15 years old).

**Intervention and comparator:**

PRoGRAM‐A (Preventing Gambling Related Harm in Adolescents), a peer‐led social network intervention to protect young people, their friends and family members from gambling related harm (GRH). Control schools delivered their standard Personal, Social, Health and Education (PSHE) curriculum, which did not include any form of gambling education.

**Measurements:**

The primary outcome of this study was whether progression to a full‐scale Phase III cRCT was warranted, using pre‐set progression criteria. These criteria sought to address uncertainties in the intervention and cRCT design with thresholds set according to a traffic light system.

**Findings:**

All five progression criteria were met. All schools were recruited and retained in the study with minimal missing outcome data. The process evaluation indicated that PRoGRAM‐A was acceptable to multiple stakeholders and delivered with fidelity to the delivery manual. The proposed primary outcome for a future Phase III cRCT was self‐reported gambling participation (measured by asking about types of gambling participation ‘in the last 4 weeks’ and ‘in the last 12 months'). This pilot study found no statictically significant differences between the control and intervention groups at follow‐up.

**Conclusions:**

The school‐based gambling prevention intervention PRoGRAM‐A appears to be an acceptable intervention which can be delivered with high fidelity. The trial methods were acceptable with all settings recruited and retained. Progression to a larger randomised controlled trial to test effectiveness and costs effectiveness is warranted.

## INTRODUCTION

Gambling is a highly profitable commercial activity, with the predicated global gambling revenue to be approximately 700 billion dollars by 2028 [1]. Public awareness and acceptability of gambling has grown with the development of sophisticated and targeted marketing strategies contributing to the ‘normalisation’ of gambling [2, 3, 4]. Gambling is, therefore, increasingly recognised as a global public health concern, with preventing gambling‐related harm in children and young people a key priority.

Gambling‐related harm is defined as the adverse impact from gambling on the health and wellbeing of individuals, families, communities and society. Problem gambling is characterised by impaired control over gambling, substantial negative consequences from this impaired control and persistence in gambling despite these negative consequences. Observational studies of gambling‐related harms indicate that gambling participation in childhood has a negative impact on young people's finances, emotional and academic development, relationships and physical and mental health, which may extend beyond childhood and into later life [5, 6].

In the United Kingdom, the 2024 Young People and Gambling Survey, reported that 27% of 11 to 17‐year‐olds had spent their own money on gambling activity in the last 12 months, compared with 15% who reported vaping, 8% who reported smoking and 7% who reported using illegal drugs. Alcohol consumption remained the highest at 37%. This survey also found that 1.5% of young people experienced problem gambling, increasing from 0.7% in 2023 [7].

Gambling, as defined in the British Gambling Act 2005, means betting, gaming or participating in a lottery, where gaming means playing a game of chance for a prize and a prize is defined as money or ‘money's worth’. However, simulated gambling, defined as ‘games that replicate or mimic gambling activities but do not involve monetary risk’ [8], and ‘gambling‐adjacent’ video game add‐ons, such as loot boxes, skin betting and social casino games are not categorised as gambling under the British Gambling Act. This is despite them being increasingly common among younger people and often perceived by them as a form of gambling [9]. As such, the lines between video gaming and gambling are becoming progressively blurred. This, coupled with increased exposure to gambling marketing [10], has contributed to the normalisation of gambling, embedding it within the daily lives of young people.

There is a well‐established link between peer influence and adolescent risk‐taking behaviour, which includes gambling [11]. A qualitative rapid literature review conducted by Wardle (2019) cited several studies noting the powerful role that friends and peer networks have on young people's gambling behaviour [12]. There are several interventions targeting young people and other risk‐taking behaviours (such as tobacco, alcohol and drugs) delivered in the United Kingdom, but few evidence based and independently funded gambling prevention programmes. Existing gambling industry funded education/prevention interventions for young people are criticised for lacking independence, appropriate development and programme theory [13]. A recent systematic review of existing peer reviewed gambling education interventions delivered within the school setting, noted a number of limitations with regards to efficacy. Limitations have been reported in relation to poor study design and methodological flaws, inconsistent or problematic measurements (the use of different gambling related scales within surveys) and high variability between the types of instruments used to measure efficacy. Further problems with regards to the efficacy of existing educational interventions is noted with regards to variability in short term follow‐up assessments, with almost no evaluation of interventions reporting on long term follow‐ups [14]. All of the above has, therefore, led to calls for robust, independent, early intervention to protect young people from future gambling‐related harms (GRH), by delaying or preventing gambling experimentation [14, 15, 16]. In this article, we report results from Preventing Gambling Related Harm in Adolescence (PRoGRAM‐A), a gambling prevention pilot cluster randomised control trial (cRCT). Developed as per Medical Research Council (MRC) guidance [17], PRoGRAM‐A was developed and feasibility tested in one school, (MRC grant, MR/S019200/1), before scaling up to a pilot cRCT.

## METHODS

### Design

This study used a two‐arm pilot cRCT comparing the PRoGRAM‐A intervention to usual practice. The trial included an embedded process evaluation with a social network analysis component and a health economic scoping study. Detailed information regarding the study design is available in our published protocol [18]. This pilot study adheres to the Consolidated Standards of Reporting Trials (CONSORT) extensions for cluster randomised trials [19] and pilot and feasibility trials [20]. The study was conducted in Scottish state‐funded secondary schools, with no restriction on location or size. School recruitment was facilitated via an online information webinar, with invitations sent through the study team's research network. Schools were visited by the principal investigator and/or project manager to discuss the trial in more detail and to sign a memorandum of understanding outlining roles, responsibilities, timeline of intervention delivery and assessments.

### Participants

Schools (clusters) were recruited from a pool of Scottish state‐funded secondary schools, with no restrictions on school size or location. A mix of urban and rural schools, with large and small school populations, in deprived and affluent areas were recruited for this pilot study. The inclusion criteria consisted of students aged 13 to 15 years old (equivalent to secondary 3 in Scotland and years 9 and 10 in England, Wales and Northern Ireland), who provided assent to participate. Schools were excluded if they were residential or specifically for students with special educational needs. A payment of £500 was provided to each school on completion of the study to encourage retention.

### Ethics and consent

Ethical approval was granted by the Edinburgh Medical School Research Ethics Committee (Ref‐23 EMREC‐016), with local authority approval obtained for all six schools. Parents/carers were provided with information sheets and given the option to opt‐out, while students received a copy of the participant information sheet, which was discussed in class before baseline visits. Assent was obtained from students on the day of the baseline questionnaire visit.

### Procedures

Baseline data were collected in October 2023, before randomisation, with follow‐up data collected approximately 6 months later (April–June 2024). The intervention was delivered between October 2023 and April 2024. Data collection occurred in school halls under exam conditions where feasible. Structured observations of intervention delivery were conducted in two schools (one small, one large) to assess acceptability and fidelity. Focus groups (*n* = 8) and semi‐structured interviews (*n* = 20) were carried out with students, school staff, trainers, researchers, service commissioners and peer supporters' friends and family.

### Randomisation

Schools were randomised into two arms using a 2:1 ratio, with randomisation stratified by school size (small <200 students, large ≥200 students). The randomisation was completed by the Edinburgh Clinical Trials Unit after baseline data collection in October 2023. Intervention and control groups were blinded to allocation, and the trial statistician remained blinded throughout.

### Intervention

**FIGURE 1:**
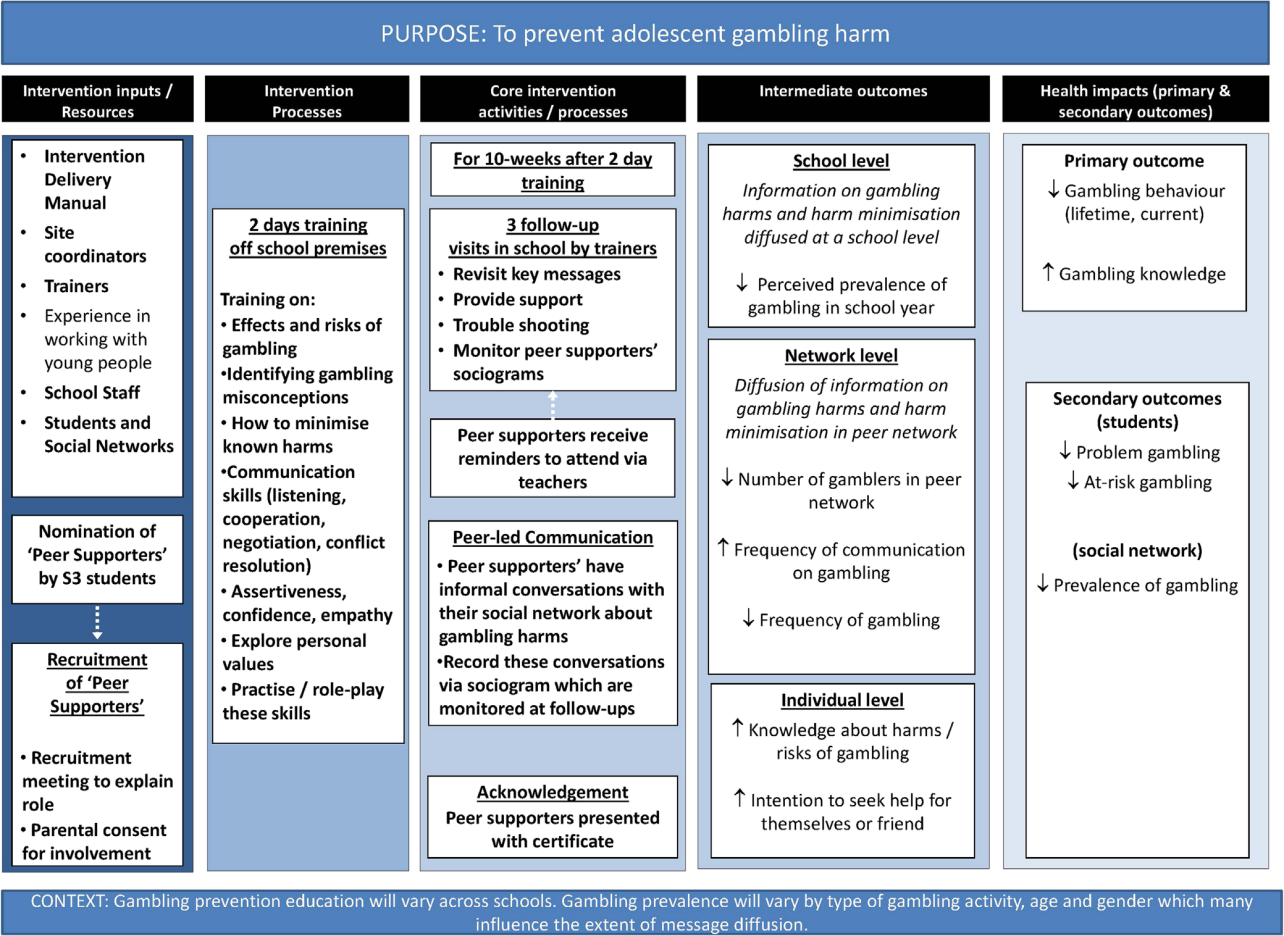
PRoGRAM‐A logic model.

The PRoGRAM‐A intervention is a peer‐led, social network intervention aimed at protecting young people from gambling‐related harm. Peer supporters, selected by their peers, participated in a two (full day) gambling education training programme, delivered away from the school setting. These peer supporters were encouraged to initiate gambling‐related conversations within their peer and family networks, tracked through social network maps (sociograms). They received three follow‐up support sessions from PRoGRAM‐A trainers. Further details on the intervention can be found in the supplementary materials (Data S2), including the TIDiER template [21] and logic model (Figure 1).

### Measures

Guided by CONSORT guidelines for pilot studies [20], the primary outcome of this pilot study was to assess whether progression to a full‐scale phase III cRCT was warranted, using pre‐set trial‐level progression criteria (Table 1). These criteria, set according to a traffic light system, addressed uncertainties in the intervention and trial design. The study also explored potential primary individual‐level outcomes for future trials, such as gambling participation (self‐reported, 4‐week and 12‐month periods), reduction in gambling harms, increased knowledge of gambling harm and reduction in positive attitudes toward gambling and gambling marketing.

**TABLE 1:** PRoGRAM‐A progression criteria.

Progression criterion	Red	Amber	Green	Actual
1. Successful recruitment of six schools	<6		6	6
2. Five schools remain in the pilot study	<4	4	≥5	6
3. The intervention being delivered with 80% fidelity to the manual	≤69%	70–79%	≥80%	95%
4. The process evaluation indicates the intervention is acceptable to students and staff	Low	Medium	High	High
5. 70% of students complete the student questionnaire at baseline and follow‐up	≤59%	60–69%	≥70%	69.2% 95% CI (67.6%–71.7%)

Abbreviation: PRoGRAM‐A, preventing gambling‐related harm in adolescents.

### Statistical analysis

Descriptive statistics were used to summarise the demographic characteristics of students and the completion rates of baseline and follow‐up questionnaires. Missing data proportions were reported. Given that this was a pilot trial, no hypothesis testing was performed. For each quantitative outcome measure, descriptive statistics were calculated. We analysed the indicative primary outcomes using a mixed effects logistic regression model with ‘self‐reported gambling at baseline’ and ‘allocation’ modelled as fixed effects and ‘school’ as a random effect OR (intervention vs. control) and 95% CI are presented alongside an intra cluster correlation (ICC). As these analyses were not informed by a sample size calculation, no *P*‐values were presented.

### Sample size calculation

As this was a pilot study, the sample size was not powered for effectiveness. The sample size was determined based on the minimum number of schools (*n* = 6) required to address the objectives, rather than a sample size calculation. This minimum number was informed by two factors. First, the size of previous pilot cRCTs of complex interventions [22, 23, 24], where it is necessary to balance the sample size needed to address uncertainties in the intervention. Second, the acceptability of trial methods against value for money of the research as a pilot study where a subsequent full scale study may be required. The number of student participants reflects the year group sizes of the recruited schools. A randomisation ratio of 2:1 was used to maximise the knowledge gained on the PRoGRAM‐A intervention, while also retaining a non‐intervention control group.

### Process evaluation

Qualitative data from interviews and focus groups were analysed through inductive thematic analysis [25] using NVivo 14. The analysis was informed by structured observations of intervention delivery to assess fidelity. A draft analytical framework was created and refined, with two members of the research team coding the data.

### Social network analysis

Sociograms were used to evaluate the scope and reach of the intervention by tracking message diffusion among peer supporters. Data were collected from the sociograms and baseline surveys, and analysed using Stata 18 [17].

### Delivery costs

Delivery costs for PRoGRAM‐A were assessed through consultation with Evidence to Impact, the organisation responsible for intervention delivery. The total programme cost per school was calculated, and the mean cost per student and peer supporter was derived.

## RESULTS

**FIGURE 2:**
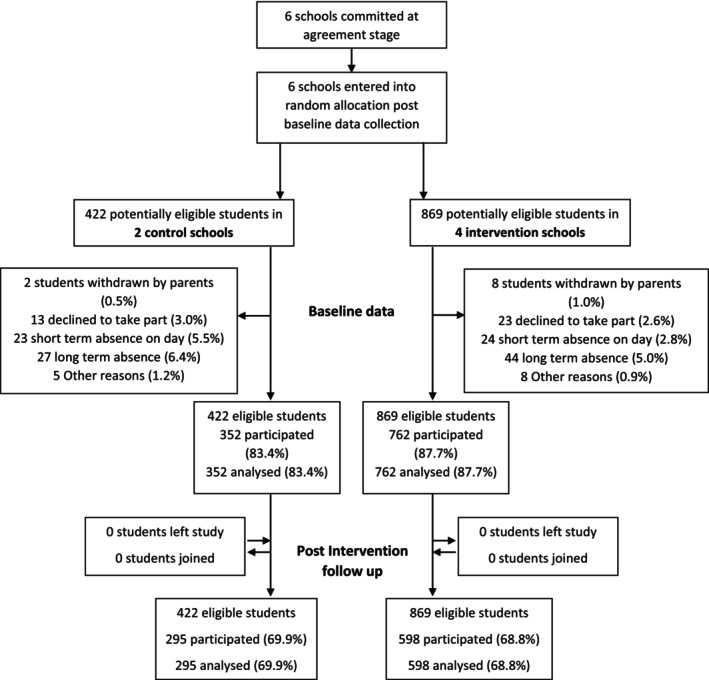
Consolidated Standards of Reporting Trials (CONSORT) flow diagram.

There were 1114 students at baseline and 893 at follow‐up (Figure 2). Of the recruited participants 45% were boys, 73% were 14 years old and 79% were from a white British ethnicity. Free school meals were used a predicator of deprivation and 7% (*n* = 76) indicated that they received free school meals or a voucher for school meals (Table 2).

**TABLE 2:** Baseline student sample characteristics.

	Intervention (*n* = 762) (4 schools)	Control (*n* = 352) (2 schools)	All (*N* = 1114)
Gender, *n* (%)			
Boy	349 (46.0)	149 (42.6)	498 (44.9)
Girl	391 (51.5)	190 (54.3)	581 (52.4)
I do not want to answer	14 (1.8)	5 (1.4)	19 (1.7)
Neither word describes me	5 (0.7)	6 (1.7)	11 (1.0)
Missing	3	2	5
Age, *n* (%)			
13 y	190 (25.0)	92 (26.4)	282 (25.5)
14 y	560 (73.8)	250 (71.8)	810 (73.2)
15 y	9 (1.2)	6 (1.7)	15 (1.4)
Missing	3	4	7
Ethnicity, *n* (%)			
White British (incl. White British, English, Scottish, Nth Irish)	644 (85.2)	227 (65.2)	871 (78.9)
White not British	20 (2.6)	42 (12.1)	62 (5.6)
Asian or Asian British	35 (4.6)	34 (9.8)	69 (6.3)
Black or Black British	15 (2.0)	5 (1.4)	20 (1.8)
Mixed/multiple ethnic backgrounds	22 (2.9)	22 (6.3)	44 (4.0)
Other	20 (2.6)	18 (5.2)	38 (3.4)
Missing	6	4	10
Do you get free school meals or a voucher for free school meals? *n* (%)			
Yes	49 (6.5)	27 (7.7)	76 (6.8)
No	623 (82.1)	278 (79.0)	901 (81.1)
I do not know	87 (11.5)	47 (13.4)	134 (12.1)
Missing	3	0	3
Are any of the adults that you live with in paid work, either part‐time or full time? *n* (%)			
Yes	685 (90.5)	308 (88.3)	993 (89.8)
No	26 (3.4)	15 (4.3)	41 (3.7)
I do not know	46 (6.1)	26 (7.4)	72 (6.5)
Missing	5	3	6

### Progression criteria

Table 1 presents results of the pilot cRCT as per the pre‐set progression criteria, which determines progression to a phase III trial. All criteria were met and four were rated green.

#### Successful recruitment of six schools

Six schools were successfully recruited.

#### Five schools remain in the pilot study

All six schools were retained for the full study duration.

#### The intervention being delivered with 80% fidelity to the manual

Results from observation of intervention delivery in two schools, indicated that all aspects of the PRoGRAM‐A training manual were delivered as intended, but because of a delivery deviation noted for one of the follow‐up we report 95% adherence to the intervention delivery. Observation also indicated that social network maps were updated by all peer supporters who attended the follow‐up session.

#### The process evaluation indicates the intervention is acceptable to students and staff

Teaching staff and students were very positive about PRoGRAM‐A, suggesting that the intervention was acceptable and a welcome addition to the school. From a staff perspective, it was noted that gambling and GRH was a growing concern and a topic that was highly relevant to students. They also recognised the specific importance of countering gambling industry narratives and societal norms around gambling activities being fun. However, teachers reported not having the time to allow them to fully engage with the topic. They were, therefore, happy to have external experts deliver this work. This, combined with the minimal time required and no financial cost, made PRoGRAM‐A very appealing, as summarised by one teacher describing why their school took part:

‘If you think of how much gambling affects the majority of people's lives in one way or another and how almost accepted it is until it's too late, I think anything that increases that awareness has to be useful. And it's how do we [teachers] fit it in with everything else we're expected to [deliver within the curriculum]’

Teacher 1, control school

Similar to teachers, interest in learning more about gambling and GRH was a common factor in student decision making to become a peer supporter. This was followed by a curiosity to participate in a research study, adding their involvement to their CV and the opportunity to participate in an external training workshop away from school. Additionally, students reported feeling proud to have been nominated by their year group to become peer supporters.

### Mode of delivery

Teaching staff were enthusiastic about the delivery of PRoGRAM‐A through peer‐education. Students taking ownership and responsibility for their own learning via the role of a peer supporter was felt to be a positive feature and, with the associated social and leadership skills acquired through this process, was highly valued by teachers. The combination of the topic area alongside the peer delivery model adopted by PRoGRAM‐A were reported to be the motivating factors that contributed to the decision to participate in the trial.

Delivery of PRoGRAM‐A by external experts with youth work experience was recognised as a positive feature given their capacity to bring topics to life in a manner that teachers felt unable to replicate. Peer supporters were equally enthusiastic, viewing the trainers as more friendly than teachers and their interactions less formal. This dynamic contributed to the creation of a relaxed environment, which facilitated peer supporter engagement in activities, as one peer supporter captured:

‘I think it also helps with like the people who display [deliver] it. You might be a little more honest with a person you've never met before, or maybe a little less honest, it just depends on the person. But you might not be straight out honest with a teacher because you're going to see them the next day.’

Alasdair, peer supporter (FG1)

#### A total of 70% of students complete the student questionnaire at baseline and follow‐up

Each school provided a student class list, which served as the sample file. However, after initial visits, it became clear that these lists did not reflect the true number of students eligible to participate. Survey fieldwork identified 71 students in the sample file at baseline who had either left or were not attending school. Statistical analysis of student survey completion was, therefore, calculated in two ways. First using the original sample file (*n* = 1291), which resulted in a completion rate of 69.2%, 95% CI = 67.6%–71.7% for both baseline and follow‐up questionnaires. Second, using the amended sample file, removing the 71 students who were not eligible to take part (*n* = 1220) increased the completion rate to 73.2%, 95% CI = 70.6%–75.7%.

### Outcome data

We found low rates of missing data for almost all variables with no major differences across arms. The proposed primary outcome measures for a future phase III cRCT are self‐reported gambling participation (measured by asking about types of gambling participation ‘in the last 4 weeks’ and ‘in the last 12 months’) [18]. As shown in Table 3, the prevalence of gambling participation in the last 4 weeks at the 6‐month follow‐up was 43% in the intervention arm and 34% in the control arm (OR = 1.21, 95% CI = 0.37–2.74). The prevalence of gambling participation in the last 12 months at the 6‐month follow‐up was 81% in the intervention arm and 73% in the control arm (OR = 1.33, 95% CI = 0.45–2.22, ICC = 0.01). Secondary outcomes measures are reported in the Data S1.

**TABLE 3:** Gambling behaviour at 4 weeks and 12 months.

	Allocation		
	Intervention *n* = 762	Control *n* = 352	All *N* = 1114
Baseline			
Self‐reported gambling last 4 weeks			
Yes	367 (48%)	134 (38%)	501 (45%)
No	394 (52%)	217 (62%)	611 (55%)
Missing	1	1	2
Self‐reported gambling last 12 months			
Yes	599 (79%)	255 (73%)	854 (77%)
No	162 (21%)	96 (27%)	258 (23%)
Missing	1	1	2
Follow up			
Self‐reported gambling last 4 weeks			
Yes	258 (43%)	99 (34%)	357 (40%)
No	340 (57%)	196 (66%)	536 (60%)
Missing	164	57	221
Self‐reported gambling last 12 months			
Yes	483 (81%)	216 (73%)	699 (78%)
No	115 (19%)	79 (27%)	194 (22%)
Missing	164	57	221

*Note*: Follow‐up missing data counts include students who participated at baseline but not follow‐up (students missing at follow‐up—overall *N* = 217, control *n* = 54, intervention *n* = 163).

### Delivery costs

The estimated delivery cost of PRoGRAM‐A for two groups of up to 30 peer supporters (sharing 1 coach) in one school (based on costs from two intervention schools with a total of 296 total students and 42 peer supporters) was £8313.00. This equates to a mean delivery cost per school student of £28.08, and £197.97 per peer supporter, or £138.55 per peer supporter at the maximum of 60 peer supporters. These costs are necessarily speculative at this phase of the research and are only intended as a guiding approximation.

### Social network analysis

Analysis of the peer supporters' social network maps found that self‐reported message diffusion was high. As Table 4 shows, peer supporters predicted having 1323 conversations, but actually had 1555. They spoke, on average, to 2.4 (step) parents or siblings, 1.6 other family members and 6.9 people outside their family. They had conversations with 68% of people they predicted they would, while 42% of their discussions were with people they never predicted they would. This suggests that the intervention was widely discussed by peer supporters and that inclusion of sociograms was an effective way of enabling them to proactively target individuals within their networks. Underestimating how many people they would talk to implies that peer supporters often raised issues naturally in conversations rather than just targeting those mentioned in the training sessions.

**TABLE 4:** Message diffusion of PRoGRAM‐A, by type of relationship to peer supporter and whether conversation was predicted in training.

	Parents and siblings	Other family	Non‐family	Total
Predicted conversations	316	268	739	1323
Actual conversations	343	224	990	1555
Percentage of predicted conversations which happened	81%	46%	71%	68%
Percentage of conversations with people not nominated in training	25%	45%	42%	42%

Abbreviation: PRoGRAM‐A, preventing gambling‐related harm in adolescents.

### Intervention refinements

The process evaluation revealed several intervention refinements that could be made [26]. Students engaged well with the topic of gambling and GRH. However, some felt that gambling‐related harm would be more realistic and relatable if they were presented with real world/lived experience examples within the materials and resources delivered across the 2‐day training workshop. Future iterations of the training materials should embed some real‐world examples of gambling and gambling related harm. Peer supporters enjoyed the interactive and engaging activities within the training workshop, which took place in a venue outside of school grounds. The follow‐up sessions, however, ran within school classrooms and were time‐limited to one class period. Feedback from students suggested that follow‐up sessions were less engaging than the workshop.

Last, despite teachers being overwhelmingly positive about the planning and organisation of PRoGRAM‐A, there were challenges of coordinating pupils and liaising with other teaching staff. If progressing to a full‐scale trial, it would be beneficial to recommend the role of Link Teacher be shared between two members of staff, particularly within larger schools.

## DISCUSSION

A pilot cRCT of the PRoGRAM‐A intervention was conducted to decide whether progression to a full‐scale phase III cRCT was warranted. All five progression criteria were met. All schools were recruited and retained in the study, and there were low levels of missing data on outcomes. The process evaluation indicated that PRoGRAM‐A was acceptable to multiple stakeholders and delivered with fidelity to the delivery manual. This suggests that progression to a full scale cRCT of PRoGRAM‐A is warranted.

In a recent systematic review of gambling prevention interventions [14], all identified interventions were delivered by teachers or external gambling experts in traditional classroom settings. Moreover, this review identified a scarcity of interventions underpinned by evidence and longer‐term follow‐up. To the best of our knowledge, PRoGRAM‐A is the first gambling prevention intervention delivered through peer‐education, and one of the few to be evidence‐based.

For the progression criteria relating to the acceptability of the cRCT design, all settings were recruited and retained at follow‐up. The student level response rate at baseline and follow‐up was 69.2% (using the original sample file) and 73.2% (using the corrected sample file). Although this met our progression criteria, we only had costed for two follow‐up visits to schools. Increasing the number of fieldworkers attending each setting and making more visits are likely to increase the number of participants retained.

The proposed primary outcomes for a future phase III cRCT was self‐reported gambling participation (measured by asking about types of gambling participation ‘in the last 4 weeks’ and ‘in the last 12 months'). Pilot study findings found no indication of differences between the control and intervention groups on either the baseline or follow‐up data. This is mostly likely because of the small number of schools included in the pilot study. It is also important to note that the follow‐up period for the pilot study was 6 months, not 12 months. Therefore, the time period was not long enough to show any change. In light of this finding, the primary outcome measure for a future cRCT has been amended to be tighter focused, measuring spending of students own money on any gambling activity in the past 12 months. If we assume 28% answer yes to this outcome in the control group, a study of 58 clusters (schools) (7366 students) would be required to give 90% power to detect a 20% relative reduction (5.5% absolute reduction) compared to the intervention group, at the 5% significance level. The assumptions supporting this from the pilot are ICC = 0.01 survey response of 70% among students. Other assumptions are a 5% dropout of schools following randomisation, and coefficient of variation of 0.35 in year group size.

The process evaluation identified refinements that could be made to the PROGRAM‐A before it is tested further. These include making intervention activities more engaging by including more real‐world examples of the effects of gambling and reducing the number of follow‐ups. All are achievable refinements within the current intervention design.

### Strengths and limitations

A key strength of PRoGRAM‐A is that it is one of the first evidence based and independently funded research studies, funded by the National Institute for Health and Social Care Research (NIHR), to prevent gambling‐related harm in adolescence. Existing gambling industry funded education/prevention interventions for young people are criticised for lacking independence, appropriate development and programme theory [13].

A further strength is that PRoGRAM‐A is grounded in, and guided by, existing intervention development and evaluation guidelines [27] with consistent stakeholder input to inform manual content and delivery. We used a robust and rigorous mixed method process evaluation, and also included a social network analysis component and a health economic scoping study.

We used a robust mixed method comprising quantitative and qualitative data collection and analysis. The students, teachers and parents who took part in the focus groups and interviews were, however, a self‐selecting sample. Those who did not take part may have given different responses from those who chose to participate. That said, students and staff were open about what they did not like about the intervention. Although estimated delivery costs were only based on a single setting and are unlikely represent the true costs in a wider rollout, its component parts provide a checklist for measurement in future trials.

## CONCLUSIONS

PRoGRAM‐A is an acceptable school‐based peer‐led intervention to prevent gambling behaviour, which can be delivered with high fidelity. The trial methods were acceptable with all settings recruited and retained. Some minor refinements in the intervention and trial methods would help to address students' perception of the real‐world effects of gambling and optimise follow‐up data collection protocols before further testing in a larger trial.

## AUTHOR CONTRIBUTIONS

**Fiona Dobbie:** Conceptualisation (lead); funding acquisition (lead); methodology (equal); resources (lead); supervision (lead); writing—original draft (lead); writing—review and editing (lead). **Martine Miller:** Conceptualisation (supporting); formal analysis (lead); funding acquisition (supporting); investigation (supporting); methodology (supporting); project administration (supporting); writing—original draft (supporting); writing—review and editing (supporting). **Angela Niven:** Data curation (equal); project administration (lead); supervision (supporting); writing—original draft (supporting); writing—review and editing (supporting). **Heather Wardle:** Conceptualisation (supporting); funding acquisition (supporting); investigation (supporting); methodology (supporting); writing—original draft (supporting); writing—review and editing (supporting). **Christopher Weir:** Conceptualisation (supporting); formal analysis (lead); funding acquisition (supporting); methodology (equal); supervision (supporting); writing—original draft (supporting); writing—review and editing (supporting). **Hannah Ensor:** Formal analysis (lead); methodology (supporting); writing—original draft (supporting); writing—review and editing (supporting). **Andrew Stoddart:** Conceptualisation (supporting); formal analysis (lead); funding acquisition (supporting); methodology (supporting); writing—original draft (supporting); writing—review and editing (supporting). **David Griffiths:** Conceptualisation (supporting); data curation (supporting); formal analysis (lead); funding acquisition (supporting); investigation (supporting); methodology (supporting); visualisation (lead); writing—original draft (supporting); writing—review and editing (supporting). **Leon Noble:** Data curation (equal); formal analysis (equal); project administration (supporting); writing—original draft (supporting); writing—review and editing (supporting). **Richard Purves:** Funding acquisition (supporting); writing—original draft (supporting); writing—review and editing (supporting). **James White:** Conceptualisation (supporting); formal analysis (supporting); funding acquisition (supporting); methodology (supporting); supervision (supporting); writing—original draft (supporting); writing—review and editing (supporting).

## DECLARTION OF INTERESTS

In the last 5 years, F.D. discloses grant funding for gambling‐related projects from the Medical Research Council and the National Institute for Health and Social Research. F.D. has received payment to participate in an online focus group to help develop an awareness raising intervention to increase knowledge of the marketing strategies used by the gambling industry to promote their products. In the past 5 years, H.W. discloses grant funding for gambling‐related projects from the National Institute for Health and Social Research, Economic and Social Research Council, Wellcome Trust, Office of Health Improvements and Disparities/Public Health Scotland, Gambling Commission (including regulatory settlement funds), Gambling Research Exchange Ontario, Greater London Authority, Greater Manchester Combined Authority and the Department for Culture Media and Sport. Between 2015 and 2020, she was Deputy Chair of the Advisory Board for Safer Gambling, providing independent advice to government on gambling policy with remuneration from the Gambling Commission. She has been paid consultancy fees by the Institute of Public Health, Ireland and the National Institute for Economic and Social Research. She is a member of the World Health Organization panel on gambling. She was paid as an expert witness on gambling by Lambeth and Middlesbrough Borough Councils. She received payment for delivery of a webinar by McGill University and travel costs to deliver a Keynote Address to the Gambling Regulators European Forum. She has received travel cost from the Turkish Green Crescent Society and Alberta Gambling Research Institute. She has provided unpaid research advice to GamCare. She runs a research consultancy practice for public and third sector bodies—she has never provided consultancy services to the gambling industry. No other authors have anything to declare.

## CLINICAL TRIAL REGISTRATION

Research Registry – researchregistry8699.

## Supporting information


**Data S1.** Supplementary Information.

**Data S2.** Supplementary Information.

## Data Availability

The data that support the findings of this study are available on request from the corresponding author. The data are not publicly available due to privacy or ethical restrictions.
