# Supplementary material for: Preventing gambling‐related harm in adolescents (PRoGRAM‐A), a secondary school‐based social network intervention: Results from a pilot cluster randomised controlled trial
Source: Addiction. 2025 Dec 12;121(4):777–87. doi: 10.1111/add.70267 (PMC12980299; doi:10.1111/add.70267)
Supplement: Supplementary file 1 — Data S1. Supplementary Information. [file ADD-121-777-s001.docx]

Table S1: Gambling harm DSM-IV-MR-J at baseline and 6-month follow-up

|  | **Baseline** | | | **Follow-up** | | |
| --- | --- | --- | --- | --- | --- | --- |
|  | **Overall (n=153)** | **Control (n=44)** | **Intervention**  **(n=109)** | **Overall (n=202)** | **Control (n=54)** | **Intervention (n=148)** |
| **Non problem gambler** | 113 (73.9%) | 31(70.5%) | 82 (75.2%) | 163 (80.7%) | 44 (81.5%) | 119 (80.4%) |
| **Risk gambler** | 18 (11.8%) | 4 (9.1%) | 14 (12.8%) | 25 (12.4%) | 6 (11.1%) | 19 (12.8%) |
| **Problem gambler** | 22 (14.4%) | 9 (20.5% | 13 (11.9%) | 14 (6.9%) | 4 (7.4%) | 10 (6.8%) |

Table S2: Gambling knowledge baseline and 6-month follow-up

|  |  | **Baseline** | | | **Follow-up** | | |
| --- | --- | --- | --- | --- | --- | --- | --- |
|  |  | **Overall (n=1114)** | **Control (n=352)** | **Intervention**  **(n=762)** | **Overall (n=1114)** | **Control (n=352)** | **Intervention (n=762)** |
| **Gambling is dangerous** | Strongly Agree | 240 (22.1%) | 74 (21.8%) | 166 (22.3%) | 203 (23.0%) | 72 (24.5%) | 131 (22.3%) |
|  | Agree | 486 (44.8%) | 167 (49.1%) | 319 (42.9%) | 431 (48.9%) | 141 (48.0%) | 290 (49.3%) |
|  | Neither Agree nor Disagree | 266 (24.5%) | 74 (21.8%) | 192 (25.8%) | 182 (20.6%) | 58 (19.7%) | 124 (21.1%) |
|  | Disagree | 66 (6.1%) | 17 (5.0%) | 49 (6.6%) | 48 (5.4%) | 16 (5.4%) | 32 (5.4%) |
|  | Strongly Disagree | 26 (2.4%) | 8 (2.4%) | 18 (2.4%) | 18 (2.0%) | 7 (2.4%) | 11 (1.9%) |
|  | Missing | 30 | 12 | 18 | 232 | 58 | 174 |
| **Gambling is safer if you practice first** | Strongly Agree | 55 (5.1%) | 9 (2.7%) | 46 (6.2%) | 41 (4.6%) | 15 (5.1%) | 26 (4.4%) |
|  | Agree | 226 (20.9%) | 78 (23.0%) | 148 (19.9%) | 166 (18.8%) | 62 (21.1%) | 104 (17.6%) |
|  | Neither Agree nor Disagree | 400 (37.0%) | 121 (35.7%) | 279 (37.6%) | 288 (32.6%) | 80 (27.2%) | 208 (35.3%) |
|  | Disagree | 293 (27.1%) | 96 (28.3%) | 197 (26.5%) | 300 (33.9%) | 102 (34.7%) | 198 (33.6%) |
|  | Strongly Disagree | 108 (10.0%) | 35 (10.3%) | 73 (9.8%) | 89 (10.1%) | 35 (11.9%) | 54 (9.2%) |
|  | Missing | 32 | 13 | 19 | 230 | 58 | 172 |
| **Gambling is an easy way to make money** | Strongly Agree | 50 (4.6%) | 9 (2.7%) | 41 (5.5%) | 40 (4.5%) | 14 (4.8%) | 26 (4.4%) |
|  | Agree | 139 (12.9%) | 37 (11.0%) | 102 (13.7%) | 108 (12.2%) | 27 (9.2%) | 81 (13.8%) |
|  | Neither Agree nor Disagree | 483 (40.6%) | 124 (36.8%) | 314 (42.3%) | 318 (36.0%) | 99 (33.7%) | 219 (37.2%) |
|  | Disagree | 314 (29.1%) | 104 (30.9%) | 210 (28.3%) | 312 (35.3%) | 102 (34.7%) | 210 (35.7%) |
|  | Strongly Disagree | 138 (12.8%) | 63 (18.7%) | 75 (10.1%) | 105 (11.9%) | 52 (17.7%) | 53 (9.0%) |
|  | Missing | 35 | 15 | 20 | 231 | 58 | 173 |
| **The more you gamble, the better your chances of winning** | Strongly Agree | 56 (5.2%) | 14 (4.1%) | 42 (5.7%) | 32 (3.6%) | 10 (3.4%) | 22 (3.7%) |
|  | Agree | 88 (8.1%) | 26 (7.7%) | 62 (8.4%) | 60 (6.8%) | 21 (7.1%) | 39 (6.6%) |
|  | Neither Agree nor Disagree | 304 (28.1%) | 81 (24.0%) | 223 (30.1%) | 241 (27.3%) | 83 (28.2%) | 158 (26.8%) |
|  | Disagree | 418 (38.7%) | 132 (39.1%) | 286 (38.5%) | 360 (40.8%) | 97 (33.0%) | 263 (44.7%) |
|  | Strongly Disagree | 214 (19.8%) | 85 (25.1%) | 129 (17.4%) | 190 (21.5%) | 83 (28.2%) | 107 (18.2%) |
|  | Missing | 34 | 14 | 20 | 231 | 58 | 173 |
| **Most people my age gamble** | Strongly Agree | 25 (2.3%) | 6 (1.8%) | 19 (2.6%) | 18 (2.0%) | 8 (2.7%) | 10 (1.7%) |
|  | Agree | 37 (3.4%) | 6 (1.8%) | 31 (4.2%) | 33 (3.8%) | 10 (3.4%) | 23 (3.9%) |
|  | Neither Agree nor Disagree | 287 (26.5%) | 90 (26.5%) | 197 (26.5%) | 285 (32.4%) | 90 (30.8%) | 195 (33.2%) |
|  | Disagree | 482 (44.6%) | 165 (48.7%) | 317 (42.7%) | 388 (44.1%) | 139 (47.6%) | 249 (42.4%) |
|  | Strongly Disagree | 250 (23.1%) | 72 (21.2%) | 178 (24.0%) | 155 (17.6%) | 45 (15.4%) | 110 (18.7%) |
|  | Missing | 33 | 13 | 20 | 235 | 60 | 175 |
| **I feel well informed about the risk of gambling** | Strongly Agree | 230 (21.3%) | 55 (16.2%) | 175 (23.6%) | 191 (21.7%) | 64 (21.9%) | 127 (21.6%) |
|  | Agree | 466 (43.1%) | 163 (48.1%) | 303 (40.9%) | 431 (48.9%) | 126 (43.2%) | 305 (51.8%) |
|  | Neither Agree nor Disagree | 259 (24.0%) | 79 (23.3%) | 180 (24.3%) | 184 (20.9%) | 72 (24.7%) | 112 (19.0%) |
|  | Disagree | 78 (7.2%) | 28 (8.3%) | 50 (6.7%) | 53 (6.0%) | 21 (7.2%) | 32 (5.4%) |
|  | Strongly Disagree | 47 (4.4%) | 14 (4.1%) | 33 (4.5%) | 22 (2.5%) | 9 (3.1%) | 13 (2.2%) |
|  | Missing | 34 | 13 | 21 | 233 | 60 | 173 |
| **People have spoken to me about the potential problems that gambling can lead to** | Strongly Agree | 195 (18.1%) | 52 (15.3%) | 143 (19.3%) | 178 (20.2%) | 54 (18.5%) | 124 (21.1%) |
|  | Agree | 404 (37.4%) | 147 (43.4%) | 257 (34.7%) | 373 (42.5%) | 117 (40.1%) | 257 (43.6%) |
|  | Neither Agree nor Disagree | 214 (19.8%) | 63 (18.6%) | 151 (20.4%) | 176 (20.0%) | 63 (21.6%) | 113 (19.2%) |
|  | Disagree | 178 (16.5%) | 51 (15.0%) | 127 (17.1%) | 116 (13.2%) | 47 (16.1%) | 69 (11.7%) |
|  | Strongly Disagree | 89 (8.2%) | 26 (7.7%) | 63 (8.5%) | 37 (4.2%) | 11 (3.8%) | 26 (4.4%) |
|  | Missing | 34 | 13 | 21 | 233 | 60 | 173 |

**Note:** Follow-up missing data counts include students who participated at baseline but not follow-up (Students missing at follow-up - Overall N=217, Control N=54, Intervention N=163)

Table S3: Attitudes towards gambling baseline and 6-month follow-up

| **How strongly do you agree or disagree that it is OK for someone your age to do the following?** |  | **Baseline** | | | **Follow-up** | | |
| --- | --- | --- | --- | --- | --- | --- | --- |
|  |  | **Overall (n=1114)** | **Control (n=352)** | **Intervention**  **(n=762)** | **Overall (n=1114)** | **Control (n=352)** | **Intervention (n=762)** |
| **Try gambling to see what it is like** | Strongly Agree | 56 (5.1%) | 19 (5.5%) | 37 (5.0%) | 64 (7.2%) | 22 (7.5%) | 42 (7.1%) |
|  | Agree | 221 (20.3%) | 89 25.7%) | 132 (17.7%) | 215 (24.3%) | 67 (22.8%) | 148 (25.0%) |
|  | Neither Agree nor Disagree | 436 (40.0%) | 134 (38.7%) | 302 (40.6%) | 335 (37.9%) | 104 (35.4%) | 231 (39.1%) |
|  | Disagree | 283 (26.0%) | 78 (22.5%) | 205 (27.6%) | 222 (25.1%) | 84 (28.6%) | 138 (23.4%) |
|  | Strongly Disagree | 94 (8.6%) | 26 (7.5%) | 68 (9.1) | 49 (5.5%) | 17 (5.8%) | 32 (5.4%) |
|  | Missing | 24 | 6 | 18 | 229 | 58 | 171 |
| **Gamble once a week** | Strongly Agree | 30 (2.8%) | 8 (2.3%) | 22 (3.0%) | 26 (2.9%) | 8 (2.7%) | 18 (3.1%) |
|  | Agree | 60 (5.5%) | 20 (5.8%) | 40 (5.4%) | 38 (4.3%) | 10 (3.4%) | 28 (4.8%) |
|  | Neither Agree nor Disagree | 270 (25.0%) | 80 (23.2%) | 190 (25.8%) | 221 (25.0%) | 57 (19.4%) | 164 (27.8%) |
|  | Disagree | 450 (41.6%) | 138 (40.0%) | 312 (42.3%) | 414 (46.9%) | 141 (48.0%) | 273 (46.3%) |
|  | Strongly Disagree | 272 (25.1%) | 99 (28.7%) | 173 (23.5%) | 184 (20.8%) | 78 (26.5%) | 106 (18.0%) |
|  | Missing | 32 | 7 | 25 | 231 | 58 | 173 |
| **Gamble on special occasions (e.g. Grand National)** | Strongly Agree | 106 (9.8%) | 33 (9.5%) | 73 (9.9%) | 118 (13.4%) | 40 (13.7%) | 78 (13.3%) |
|  | Agree | 288 (26.5%) | 91 (26.3%) | 197 (26.6%) | 277 (31.5%) | 79 (27.0%) | 198 (33.7%) |
|  | Neither Agree nor Disagree | 351 (32.3%) | 114 (32.9%) | 237 (32.0%) | 269 (30.6%) | 78 (26.6%) | 191 (32.5%) |
|  | Disagree | 241 (22.2%) | 74 (21.4%) | 167 (22.6%) | 162 (18.4%) | 75 (25.6%) | 87 (14.8%) |
|  | Strongly Disagree | 100 (9.2%) | 34 (9.8%) | 66 (8.9) | 54 (6.1%) | 21 (7.2%) | 33 (5.6%) |
|  | Missing | 28 | 6 | 22 | 234 | 59 | 175 |

**Note:** Follow-up missing data counts include students who participated at baseline but not follow-up (Students missing at follow-up - Overall N=217, Control N=54, Intervention N=163)

Table S4: Attitudes towards gambling marketing baseline and 6-month follow-up

|  | **Baseline** | | | **Follow-up** | | |
| --- | --- | --- | --- | --- | --- | --- |
|  | **Overall (n=1114)** | **Control (n=352)** | **Intervention**  **(n=762)** | **Overall (n=1114)** | **Control (n=352)** | **Intervention (n=762)** |
| **I like gambling adverts a lot** | 27 (2.6%) | 9 (2.7%) | 18 (2.5%) | 32 (3.7%) | 11 (3.8%) | 21 (3.6%) |
| **I like gambling adverts a little** | 56 (5.3%) | 17 (5.1%) | 39 (5.4%) | 58 (6.6%) | 19 (6.5%) | 39 (6.7%) |
| **I neither like nor dislike gambling adverts** | 557 (52.8%) | 172 (52.0%) | 385 (53.3%) | 465 (53.1%) | 133 (45.5%) | 332 (56.9%) |
| **I dislike gambling adverts a little** | 206 (19.5%) | 65 (19.6%) | 141 (19.5%) | 186 (21.3%) | 72 (24.7%) | 114 (19.6%) |
| **I dislike gambling adverts a lot** | 208 (19.7%) | 68 (20.5% | 140 (19.4%) | 134 (15.3%) | 57 (19.5%) | 77 (13.2%) |
| **Missing** | 60 | 21 | 39 | 239 | 60 | 179 |

**Note:** Follow-up missing data counts include students who participated at baseline but not follow-up (Students missing at follow-up - Overall N=217, Control N=54, Intervention N=163)
