# Supplementary figures and images for: Preventing gambling‐related harm in adolescents (PRoGRAM‐A), a secondary school‐based social network intervention: Results from a pilot cluster randomised controlled trial
Source: Addiction. 2025 Dec 12;121(4):777–87. doi: 10.1111/add.70267 (PMC12980299; doi:10.1111/add.70267)

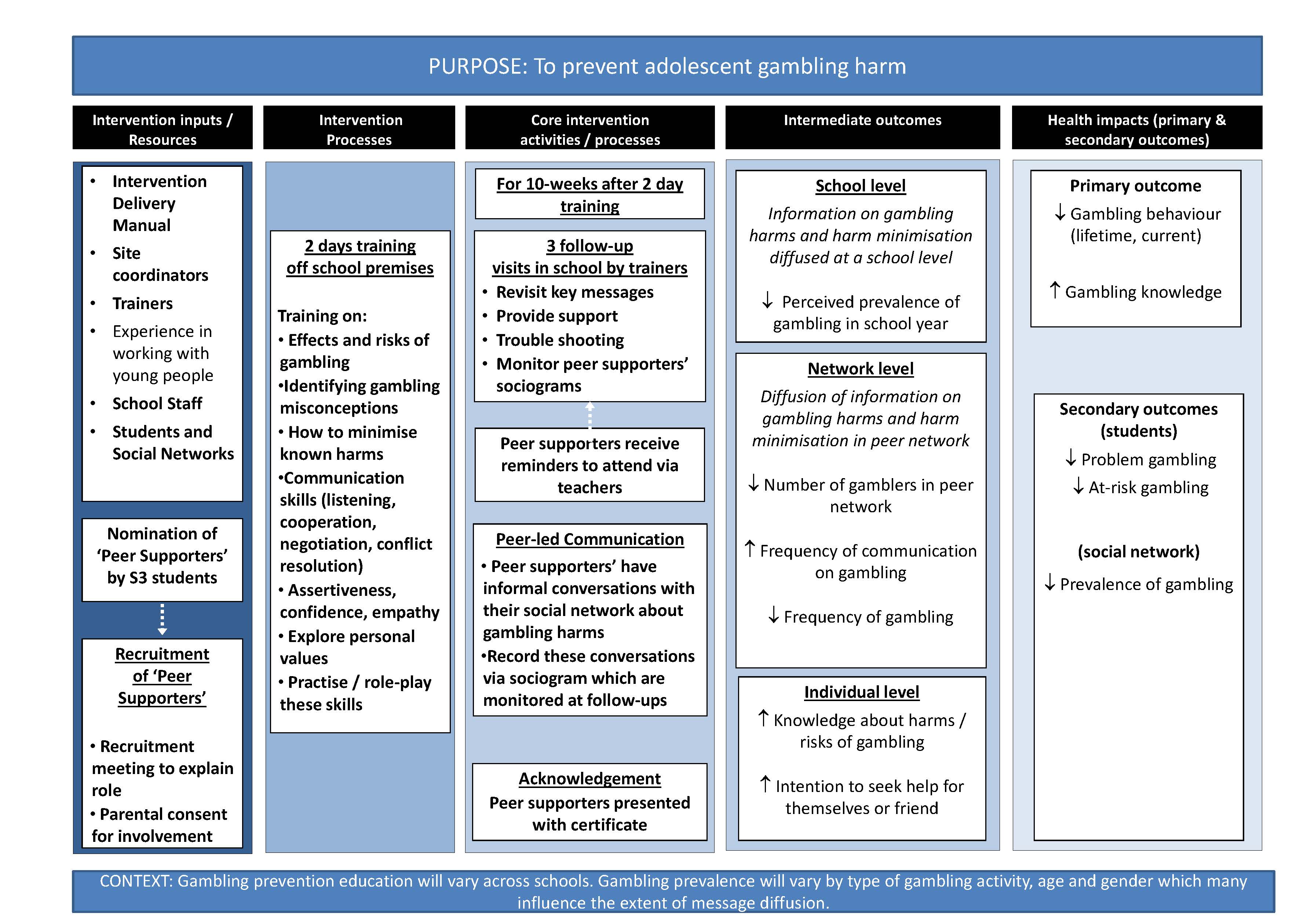

Supplement: Supplementary file 2 — Data S2. Supplementary Information. [file ADD-121-777-s002.zip › Figure 2_PRoGRAM-A Logic Model_Supp.jpg]
